# Could Different Doses of Dexmedetomidine Be as Effective as Amifostine Against Radiotherapy-Induced Liver Injury in Rats? Evidence from Mitotic, Apoptotic, Oxidative, and Neurogenic Insights

**DOI:** 10.3390/jcm14228238

**Published:** 2025-11-20

**Authors:** Hatice Beyazal Polat, Hamit Yilmaz, Kasım Demir, Kagan Kilinc, Belemir Gülhan, Sema Yilmaz Rakici, Levent Tumkaya

**Affiliations:** 1 Department of Internal Medicine, Faculty of Medicine, Recep Tayyip Erdogan University, Rize 53100, Türkiye; 2 Department of Biophysics, Faculty of Medicine, Kahramanmaraş Sütcü İmam University, Kahramanmaras 46040, Türkiye; 3 Division of Gastroenterology, Department of Internal Medicine, Training and Research Hospital, Samsun 55220, Türkiye; kasimdemir36@yahoo.com; 4 Department of Genetic and Bioengineering, Faculty of Engineering and Natural Sciences, Gumushane University, Gumushane 29000, Türkiye; kagankilinc@gumushane.edu.tr; 5 Department of Histology and Embryology, Faculty of Medicine, Samsun University, Samsun 55080, Türkiye; gulhan.belemir@gmail.com; 6 Radiation Oncology, Recep Tayyip Erdogan University, Rize 53100, Türkiye; sema.rakici@erdogan.edu.tr; 7 Department of Histology and Embryology, Faculty of Medicine, Ondokuz Mayıs University, Samsun 55139, Türkiye; levent.tumkaya@omu.edu.tr

**Keywords:** dexmedetomidine, amifostine, apoptotic index, radiotherapy, liver, GAP-43

## Abstract

**Background/Objectives**: Radiotherapy (RT) induces oxidative stress and structural damage in solid tissues, including the liver. This study aimed to investigate the histological and immunohistochemical effects of dexmedetomidine (DEX) and amifostine on their potential protective and regenerative properties against liver injury induced by radiation therapy. **Methods**: This study consisted of five randomized groups: control, RT, RT-D100, RT-D200, and RT-A (Amifostine). A total of 100 µg/kg DEX, 200 µg/kg DEX, and 200 µg/kg amifostine were administered before radiotherapy as per the experimental design. After RT, liver specimens were analyzed for histological alterations, including periportal and perisinusoidal fibrosis, vacuolization, and pyknotic nuclei. Furthermore, immunohistochemistry investigations were conducted to evaluate apoptosis, mitosis, oxidative stress, and neural regeneration in non-neuronal liver tissue following radiotherapy and subsequent treatment. **Results**: The control group’s liver tissue exhibited standard histological architecture, whereas the RT group displayed severe cellular degeneration, periportal and perisinusoidal fibrosis, cytoplasmic vacuolization, and an increase in pyknotic nuclei. The apoptotic index was markedly reduced in the RT-D100 and RT-D200 groups relative to the RT group. Furthermore, caspase-3 immunoactivity was negligible in the control group, while a significant increase was observed in the RT group. The administration of amifostine significantly increased GAP-43 levels. **Conclusions**: DEX mitigates RT-induced hepatic injury chiefly through antioxidant and anti-apoptotic pathways, whereas amifostine promotes hepatic regeneration by modulating GAP-43.

## 1. Introduction

Radiotherapy, a primary method for treating malignant tumors in the thoracic and abdominal regions, often has its therapeutic efficacy limited by the damage inflicted on healthy tissues not intended as targets. The liver’s extensive vascularity and metabolic activity render its tissue particularly susceptible to radiation-induced damage. Acute or chronic radiation-induced liver damage might involve a variety of complex mechanisms, including oxidative stress, inflammation, apoptosis, fibrosis, and delayed tissue regeneration. These pathological alterations may worsen liver function, raise patient morbidity, and hinder the effectiveness of treatment [1,2].

Oxidative damage is a principal mechanism underlying radiation-induced tissue injury. Ionizing radiation elevates reactive oxygen species (ROS), induces lipid peroxidation, and diminishes the effectiveness of intrinsic antioxidant systems. In experimental studies, oxidative stress was significantly reduced in rats administered dexmedetomidine, while levels of antioxidant enzymes remained stable [2,3]. Apoptosis is another significant process involved in liver injury. Caspase 3, a principal executor in the final stage of apoptosis, is increased in most liver injury models. PTEN (phosphatase and tensin homolog) is a crucial protein that modulates apoptosis by suppressing the PI3K/Akt signaling pathway, consequently facilitating cellular death. Dexmedetomidine exhibits cytoprotective properties by inhibiting apoptosis in liver ischemia–reperfusion (IR) models. The effects are primarily mediated via the PI3K/Akt pathway [3,4]. Dexmedetomidine alleviates oxidative stress [5].

Fibrosis, especially in the context of chronic liver injury, is regarded as a significant factor. TGF-β3, an essential regulator of fibrogenesis, is proposed to activate hepatic stellate cells by enhancing extracellular matrix accumulation. The literature on the function of TGF-β3 in radiation therapy-induced fibrosis is insufficient and necessitates additional research [6]. Additionally, Ki-67 acts as a prominent marker for cellular proliferation, utilized to assess liver regeneration and indicate the regenerative capacity of hepatocytes. Dexmedetomidine has been shown to enhance the liver’s regenerative response by increasing Ki-67 positivity in models of partial hepatectomy. The interaction between α2-adrenergic receptors and immune cells may mediate this effect [7].

Apoptosis, proliferation, stress, and neurogenic responses all contribute to liver injury. The expression of markers like c-Fos signifies tissue stress in liver tissue [8]. Additionally, in certain conditions, such as liver regeneration, particularly regarding nerve fiber regeneration, there is an increase in GAP-43 [9]. Limited data indicate that S100β is highly expressed in hepatocytes or biliary tract cells within normal liver tissue [10]. Considering the expression of S100β in conjunction with histopathological findings in the RT-induced liver injury model is essential.

Amifostine, used in our study for its antioxidant qualities, will be examined for its potential cytoprotective effects during radiotherapy. The active metabolite, WR-1065, mitigates DNA damage by scavenging free radicals. Nonetheless, its clinical application is constrained by adverse effects, including nausea and hypotension [1,11]. Dexmedetomidine (DEX), designated for our study, is a selective α2-adrenergic receptor agonist, noted for its sedative and analgesic characteristics. Experimental studies suggest that DEX exhibits antiapoptotic, anti-inflammatory, and antioxidant properties. In models of radiation-induced liver injury, DEX diminishes malondialdehyde (MDA) levels and preserves the activity of antioxidant enzymes [2]. It is believed to be crucial in liver regeneration [3,5,12]. Based on this, investigating the effectiveness of DEX administered at different doses in preventing radiotherapy-induced liver injury compared to amifostine will provide different perspectives at the therapeutic level. In our current study, we will highlight cellular effects at the molecular level, considering fibrosis (TGF-β3), apoptosis (Caspase-3, PTEN), proliferation (Ki-67), and neurogenic/stress response (GAP-43, c-Fos, S100β) in the RT model. The primary goal of this study was to demonstrate whether DEX at different dosages is as effective as amifostine in reducing RT-induced liver damage through thorough immunohistochemical analyses.

## 2. Materials and Methods

### 2.1. Experimental Designs, Rats, and Ethical Approval

Forty male Sprague Dawley rats, weighing 350–390 g and ranging in age from 4 to 5 months, were used in the current investigation. The rats were obtained from the Recep Tayyip Erdoğan University Experimental Research Center and housed in cages with free access to tap water and standard pellet chow, maintained under a 12 h light/dark cycle, 55–60% humidity, and 22 ± 2 °C. This study was conducted using liver cadaveric tissues obtained from the research approved by the Recep Tayyip Erdoğan University Animal Experimentation Ethics Committee (Rize, Türkiye; Decision No: 2021/21, date: 2 December 2021) gave its approval to our study. Using a computer program, the rats were randomly divided into five groups (*n* = 8). Physiological saline (1 mL) was given to the control group. The RT (radiotherapy), RT + dexmedetomidine 100 µg (Dex100), RT + dexmedetomidine 200 µg, and RT + amifostine (AMF) groups were all exposed to radiation. However, 30 min prior to RT, the treatment groups received intraperitoneal injections of 200 µg/kg amifostine, 100 µg/kg dexmedetomidine, or 200 µg/kg dexmedetomidine, depending on the group.

### 2.2. Radiotherapy

Applications of radiation were carried out at Recep Tayyip Erdoğan University’s Faculty of Medicine’s Department of Oncology. Rats were given 5 mg/kg xylazine (Rompun, Bayer, Turkey) and 50 mg/kg ketamine (Ketalar, Pfizer, Istanbul, Turkey) intraperitoneally prior to the application. Rats under deep anesthesia were put in the prone position, and the CMS Xio (v5.0; Elekta, Stockholm, Sweden) system was used to perform conformal planning. An Elekta Synergy linear accelerator was used to deliver an 8 Gy dose of external irradiation in a single fraction at a distance of 100 cm and 6 MV. To examine the consequences of radiation-induced acute hepatotoxicity, the rats were killed 24 h after irradiation while under high-dose anesthesia. In the experimental model, a single dose of 8 Gy of ionizing radiation was applied to the entire body (total body irradiation) of anesthetized rats to induce liver damage.

### 2.3. Paraffin Sections, Tissue Processing, and Scoring

Following the sacrifice of the rats, liver tissues were promptly excised and preserved in 10% neutral formalin for 10 days. Subsequent to fixation, the tissues underwent conventional histological processing procedures. The liver specimens underwent dehydration through a sequential series of alcohols, were cleared with xylene, and subsequently embedded in paraffin blocks. Sections measuring 5 µm in thickness were obtained from paraffin-embedded tissues utilizing a microtome (Leica RM 2125RT, Nussloch, Germany). The resulting sections were stained with hematoxylin and eosin for histopathological evaluation. Histopathological evaluation included the presence of periportal fibrosis, perisinusoidal fibrosis, vacuolization, and pyknotic nuclei. Under a light microscope, each parameter was semi-quantitatively assessed on a scale of 0–3 (0: standard structure, 1: mild change, 2: moderate change, 3: severe change) based on the severity of tissue damage.

### 2.4. Biochemical Analyses

Liver tissues were extracted for biochemical assays, then carefully cleaned with physiological saline and kept at −80 °C until the analysis. A homogenization buffer (20 mM sodium phosphate and 140 mM potassium chloride; pH = 7.4) was used to prepare the tissues. 1 mL of buffer was added to the 100 mg sample to homogenize it. At 4 °C, the mixture was then centrifuged at 800× *g* for 10 min, and the supernatant was separated for biochemical analysis. TBARS (Thiobarbituric Acid Reactive Substances) analysis was performed using a modified version of the method described by Ohkawa et al. (1979) to determine the levels of lipid peroxidation [13]. After adding SDS, TBA, and acetic acid solutions to the samples, the incubation process was completed in a boiling water bath for one hour. A wavelength of 532 nm was used to assess absorbance in cooled samples, and the results were reported as nanomoles per gram of tissue. Ellman’s reagent (DTNB) was used to spectrophotometrically measure the yellow complex generated by free sulfhydryl groups, allowing for the calculation of total thiol Alan levels [14]. After adding DTNB and Na_2_HPO_4_ buffer to the supernatant and standards, the absorbance of the mixtures was measured at 412 nm. The results were computed as mM/g tissue. Thermo Scientific Multiskan GO (Vantaa, Finland) was used for all spectrophotometric analyses.

### 2.5. Immunohistochemical Analyses

Immunohistochemical analysis was performed on 4-µm-thick sections taken from the paraffin blocks using the HRP/AEC Mouse Detection Kit (AB93705, Abcam, Cambridge, MA, USA). Anti-TGF-β3 (Abcam, USA), anti-S100-β (Abcam, USA), anti-GAP-43 (Abcam, USA), anti-c-Fos (Santa Cruz Biotechnology, Inc., Dallas, TX, USA), anti-PTEN (Santa Cruz Biotechnology, Inc., Dallas, TX, USA), anti-Ki-67 (Abcam, USA), and anti-caspase-3 (Abcam, USA) primary antibodies. Immunohistochemical analysis was performed on four µm-thick sections taken from the same blocks using the HRP/AEC Mouse Detection Kit (Abcam, USA). Evaluation was performed with TGF-β3, S100-β, GAP-43, c-Fos, anti-PTEN, anti-Ki-67, and anti-caspase-3 primary antibodies. Mayer’s Hematoxylin was used for counterstaining. The following scale was used to evaluate immunohistochemical staining based on cytoplasmic and nuclear positivity and percentage of positive cells: The scoring system: 0 = absent, 1 = weak, 2 = moderate, 3 = strong. The evaluation was performed blindly by a histologist. This evaluation method is based on semi-quantitative immunohistochemical analysis systems.

Sections stained with Ki-67 and caspase-3 were covered with water-based coverslips and examined using a camera-mounted light microscope (Olympus, BX43, Corporate Parkway Center Valley, PA, USA) with the computer-aided cellSens Entry microscope software program (version 1.3, Olympus, Center Valley, Corporate Parkway, USA). Five sections from each animal were evaluated for each group. Areas were randomly selected for apoptotic analysis, and at least 1000 cells were counted. Apoptotic cells were assessed using caspase-3-positive cells and the following formula:

AI (%) = (apoptotic/total cell count) × 100.

The following formula was used to assess mitotic cells that stained positive for Ki-67:

MI (%) = (mitotic cell count/total cell count) × 100

**Figure 1:**
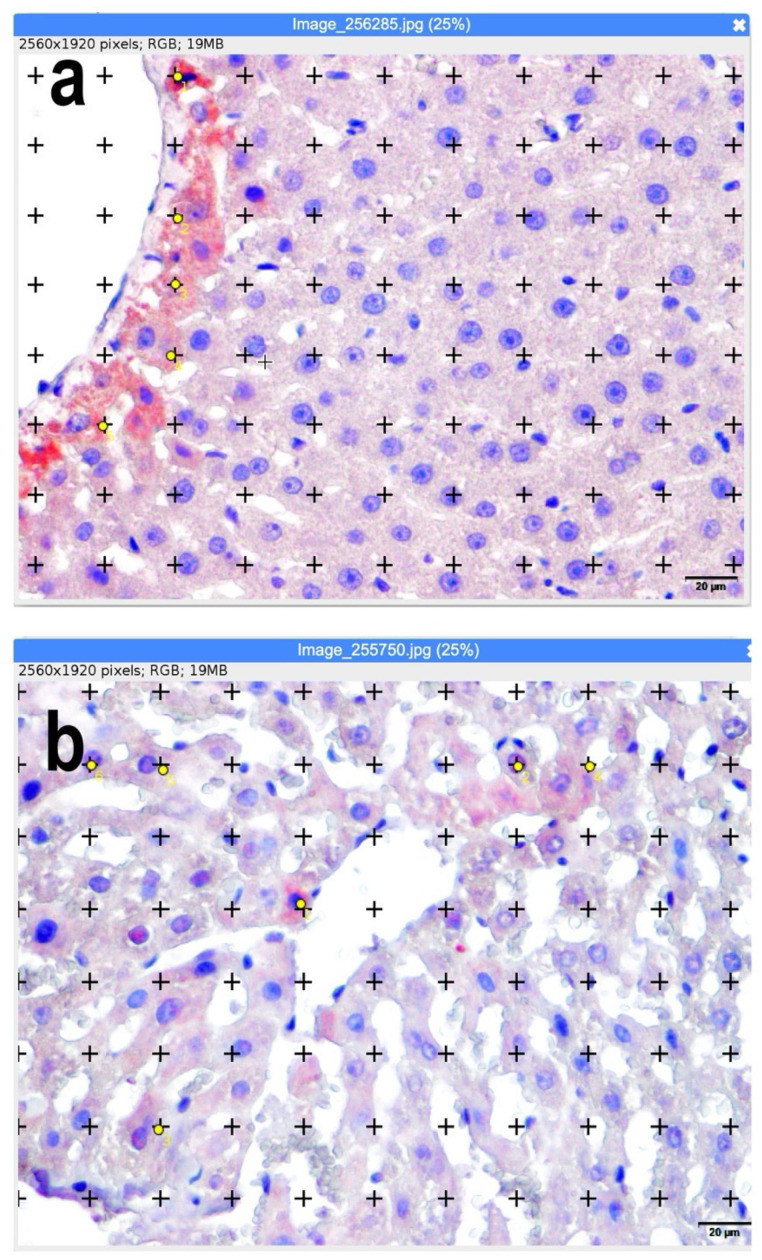
(**a**,**b**) Calculation of apoptotic and mitotic index using ImageJ software (ImageJ.JS software (version 1.53m, National Institutes of Health, Bethesda, MD, USA). Representative sections superimposed on the dot grid for volume fraction calculation are shown. Yellow dots represent apoptotic hepatocytes identified by immunohistochemical criteria. Positive staining cells with yellow points are counted. Bars: 20 µm.

ImageJ (version 1.53, National Institutes of Health, Bethesda, MD, USA) was used to analyze count areas and percentage values (Figure 1).

### 2.6. Statistics

One-way ANOVA and the Kruskal–Wallis test were used for numerical and non-parametric statistical analysis, respectively. Post hoc tests (Tukey’s for one-way ANOVA and Dunn’s for Kruskal–Wallis) with the Bonferroni correction were performed when groups differed significantly. A *p*-value < 0.05 indicated statistical significance. Each experimental group had eight rats.

## 3. Results

### 3.1. Histopathological Scoring: Histopathological Evaluation

**Figure 2:**
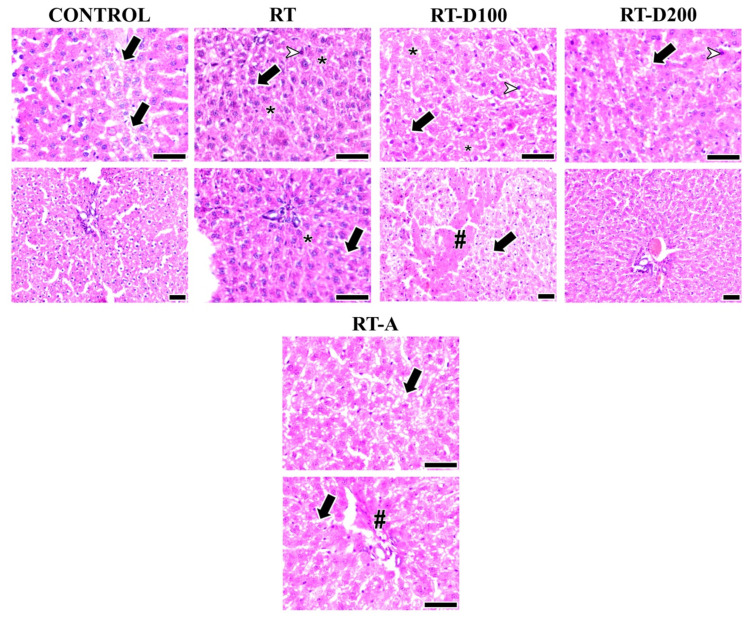
Liver images from all groups are shown. The arrow shows vacuolization. Arrowheads indicate hepatocytes with pyknotic nuclei. Intracellular spaces and degeneration (*) are observed in the RT and RT-D100 groups. Periportal fibrosis (#) is observed in the RT-A group, while perisinusoidal fibrosis (#) is observed in the RT-D100 group: hematoxylin and eosin staining; scale bars: 50 µm.

Periportal fibrosis, perisinusoidal fibrosis, vacuolization, and the presence of pyknotic nuclei were all graded semi-quantitatively and statistically in the histological evaluation of liver tissues. Only between the RT-A and RT groups was there a significant difference in periportal fibrosis. Periportal fibrosis was greater in the RT-A group than in the RT group (*p* = 0.0005). However, other group comparisons showed no significant differences (*p* > 0.05). The RT-D100 group exhibited noticeably more perisinusoidal fibrosis than the control group (*p* = 0.0025) and the RT group (*p* = 0.0026). Additionally, compared with the RT-D100 group, perisinusoidal fibrosis was significantly less in the RT-D200 group (*p* = 0.0026). RT-D200 showed no significant variation in vacuolization compared to the control group (*p* = 0.0198), whereas RT exhibited a marked increase (*p* = 0.0071). Additionally, the RT-A group showed a substantial decrease in vacuolization as compared to the RT group (*p* = 0.0102) and the RT-D200 group (*p* = 0.0277). When images from each group were evaluated for pyknotic nucleus, a decrease was found in the RT-A group compared to the RT and RT-D100 groups (*p* = 0.0001 and *p* = 0.0032, respectively). The comparisons between the other groups showed no significant differences in terms of pyknotic nucleus (*p* > 0.05) (Table 1, Figure 2).

### 3.2. Biochemical Findings

***TBARS:***

**Figure 3:**
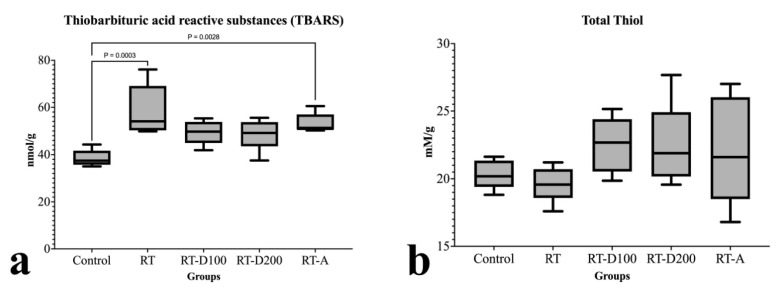
(**a**,**b**) Graphs of biochemical parameters.

Following radiation therapy [12], there was a significant increase in the TBARS levels in liver tissue when compared to the control group (*p* = 0.0003). Both low- and high-dose dexmedetomidine administrations (RT-D100 and RT-D200) significantly reduced TBARS levels compared to the RT group; however, only the difference in the RT-D100 group from the control group was statistically significant (*p* = 0.0028). TBARS levels in the RT-A group were somewhat lower than in the RT group; however, this difference was not statistically significant (Figure 3).

***Total Thiol (T-T):***

When the groups were evaluated in terms of total thiol, no difference was observed between the RT group and the control group (*p* > 0.05). Similarly, no difference was observed between the other groups (Figure 3).

### 3.3. Immunohistochemical Analyses

***TGF-β3:***

**Figure 4:**
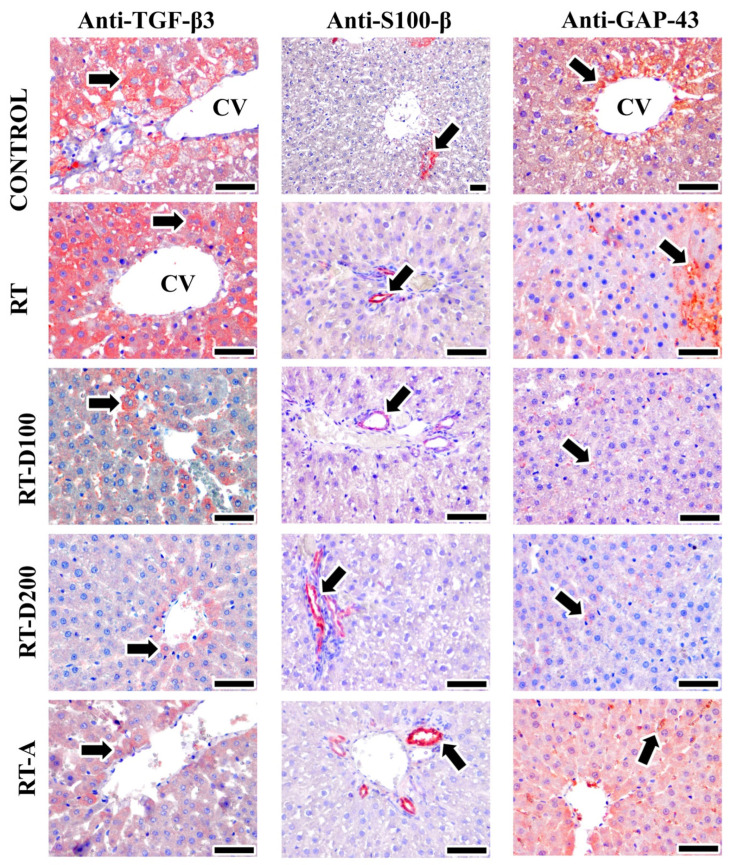
Image of positive immunoreactivity (arrow) for TGF-β3, S100-β, and GAP-43 antibodies in liver tissues from all groups. TGF-β3 reactions were high in the RT and control groups, while moderate staining was observed in the RT-D100, RT-D200, and RT-A groups. S100-β positive areas were observed to be the same in all groups, while GAP-43 positive areas were intensely stained in the Control and RT-A groups and moderately stained in the RT group. On the other hand, low-intensity positive reactions were observed in the RT-D100 and RT-D200 groups. Mayer’s hematoxylin was used for counterstaining—scale bars: 50 µm.

No significant difference in TGF-β3 levels was observed between the RT and control groups (*p* > 0.05). A significant reduction was noted in the RT-D100 group compared to the control group (*p* = 0.0124). The RT-A group exhibited significantly reduced levels of TGF-β3 compared to the control group (*p* = 0.0044). The TGF-β3 immunoreactivity in the RT group was significantly greater than in the RT-D100, RT-D200, and RT-A groups (*p* < 0.05) (Figure 4).

***Caspase-3:***

**Figure 5:**
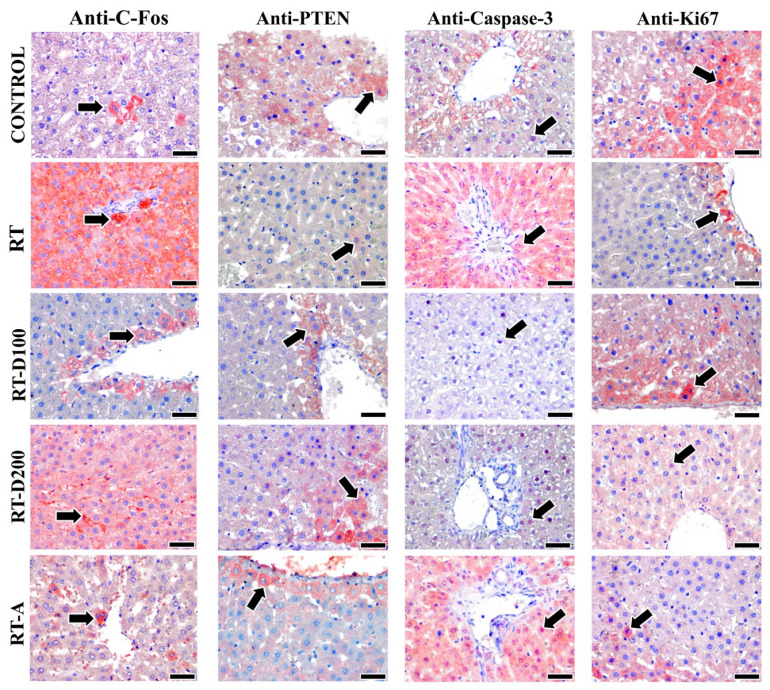
Images of positive immunoreactivity (arrow) for c-Fos, PTEN, and caspase-3 antibodies in liver tissues from all groups are shown. C-Fos antibody is observed to exhibit a more intense reaction in the RT and RT-D200 groups. It is noted that the PTEN reaction is intense in the control group and significantly lower in the RT group. Caspase-3 antibody is expressed at a high intensity in the RT and RT-A groups, while staining is observed at a lower intensity in the RT-D100 and RT-D200 groups. Furthermore, the Ki-67 antibody is observed to exhibit high-intensity staining in the Control and RT-D100 groups. However, Ki-67 antibody staining is observed at a lower intensity in the other groups. Mayer’s hematoxylin is used for counterstaining—scale bars: 40 µm.

Caspase-3 expression in the RT group exhibited a tendency to increase relative to the control group, although this finding was not statistically significant (*p* > 0.05). The RT-D100 and RT-D200 groups showed significantly reduced caspase-3 immunoreactivity to the RT group (*p* = 0.0048 and *p* = 0.0042, respectively). Furthermore, the RT-A group demonstrated increased caspase-3 expression in comparison to the RT-D100 and RT-D200 groups (*p* < 0.05) (Figure 5).

***PTEN:***

No statistically significant variation in PTEN immunoreactivity was observed between the groups (*p* > 0.05). This indicates that the administered treatment levels did not significantly impact the PTEN signaling pathways (Figure 5).

***GAP-43:***

The RT group exhibited a tendency for decreased GAP-43 expression relative to the control group; however, this difference did not reach statistical significance (*p* > 0.05). The RT-D100 and RT-D200 groups exhibited a significant reduction compared to the control group (*p* = 0.0260 and *p* = 0.0100). Furthermore, the RT-A group exhibited significantly higher expression of GAP-43 compared to the RT-D100 and RT-D200 groups (*p* < 0.01) (Figure 4).

***S100-β:***

Post-irradiation, S100-β expression showed a slight increase; however, this variation was not statistically significant (*p* > 0.05) compared to the control group. No significant differences were observed between the RT-D100, RT-D200, and RT-A groups when compared to the RT group (*p* > 0.05) (Figure 4).

***C-Fos:***

After radiation exposure, c-Fos expression significantly increased compared with the control group (*p* = 0.0033). The increase in the RT-D100 and RT-D200 groups was significantly lower (*p* < 0.05). The c-Fos levels in the RT-A group were significantly lower than those in the RT group (*p* = 0.0012). The RT-A group exhibited a significant reduction relative to the RT-D200 group (*p* = 0.0079) (Figure 5).

***Ki-67:***

The RT group exhibited a significantly reduced count of Ki-67-positive cells compared to the control group (*p* = 0.0276). In the RT-D100 group, there was an increase in proliferative activity, which was subsequently reversed (*p* = 0.0370). The RT-D200 group demonstrated a significant reduction relative to the control group (*p* = 0.0086). A significant difference was observed between the RT-D200 and RT-D100 groups (*p* = 0.0118) (Figure 5).

### 3.4. Apoptotic and Mitotic Index

**Figure 6:**
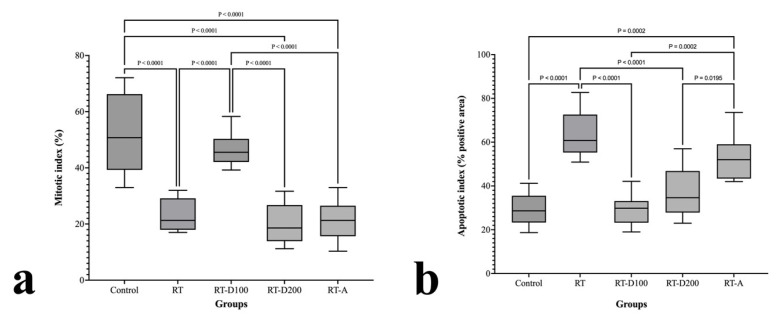
(**a**,**b**) Graphs of mitotic and apoptotic index.

The RT group was considerably greater than the control group (*p* < 0.0001) in the mitotic index (MI) assessment (Figure 6a). In contrast to the RT and RT-D200 groups, the MI in the RT-D100 group increased somewhat but stayed below the lower values. The MI was considerably higher in the RT-A group than in the RT and RT-D200 groups, but it fell short of the control group (*p* < 0.0001).

The RT group had a considerably higher apoptotic index (AI) rating than the control group (*p* < 0.0001) (Figure 6b). In contrast to the RT group, the RT-D100 group’s AI was significantly lower (*p* < 0.0001), although the AIs of the RT-D200 and RT-A groups were marginally higher. While still lower than the RT group, the AI in the RT-A group was considerably higher than that in the RT-D200 group (*p* = 0.0195). While the interventions (D100, D200, and A) slightly mitigated this impact, RT delivery was found to generally promote apoptosis.

## 4. Discussion

Radiation therapy employed for the treatment of malignant tumors may induce inflammation, oxidative stress, cellular damage, and apoptotic processes in solid organs, including the liver, resulting in cytotoxic effects. The liver, characterized by its extensive metabolic functions and significant blood supply, is notably vulnerable to toxic substances. Following radiation exposure, hepatocytes, endothelial cells, and sinusoidal structures exhibit numerous morphological abnormalities, elevated levels of intracellular reactive oxygen species, lipid peroxidation, and DNA damage [14]. According to a recent study, DEX protects the spleen, one of the solid organs, from RT-induced toxicity. At the histopathological and immunohistochemical levels, administration of 100 and 200 µg/kg DEX has shown protective effects against RT-induced damage [15]. Administration of 100 and 200 µg/kg DEX has demonstrated protective effects on RT-induced damage at both histopathological and immunohistochemical levels [16]. Similarly, when acute damage was examined after total body radiation (6 Gy) in the mouse liver, it was reported that DEX showed cytoprotective activity by reducing serum ALT/AST, TNF-α, IL-1β, ROS, and MDA levels and by activating the antioxidant mechanism [15]. Moreover, the clinical significance of DEX indicates that its application in the liver is associated with reduced levels of alanine aminotransferase and lactate dehydrogenase (LDH), as well as expedited functional recovery during the postoperative phase [17]. Cell death occurs as a result of lipid peroxidation and DNA damage caused by increased intracellular ROS due to radiation. DEX’s protective mechanism may be closely related to its antioxidant properties. In this context, it is known to lower ROS levels and activate antioxidant processes, such as total thiol [16].

In our study, the apoptotic and mitotic effects observed in the liver after RT provide evidence of a possible balance between damage and regeneration. When examining the groups administered DEX and amifostine, the 100 mg/kg group showed a more pronounced increase in mitotic activity and a decrease in apoptotic activity compared to the other groups. It is particularly noticeable that amifostine did not affect mitotic and apoptotic effects. The increased apoptotic effect resulting from RT therapy is a significant finding supporting liver damage. Caspase-3 is a critical protease that plays a key role in the final stage of the cellular apoptotic process and is regarded as a reliable marker of programmed cell death. Activated Caspase-3 contributes to the manifestation of pyknosis [18]. Radiation induces Caspase-3 activity in liver tissue. A significant increase in Caspase-3 expression was found in hepatocytes following 8 Gy radiation exposure in rats. Mitochondrial dysfunction, rather than oxidative stress, has been proposed as the trigger for this increase [14]. In a liver IR model, DEX administration reduced Caspase-3 expression; this effect was mediated by the activation of the PI3K/Akt pathway, which is known as the antiapoptotic pathway [3]. The level of PTEN, an important tumor suppressor gene/protein associated with cell growth, survival, and DNA damage repair, may vary depending on the radiation dose [19]. PTEN suppression is thought to induce Caspase 3 activity and thereby affect apoptosis [20,21]. Considering the PTEN expression levels in our study, radiotherapy may suppress PTEN and induce apoptosis. We suggested that increased PTEN activation in the antioxidant groups, combined with targeted therapy using DEX and amifostine, may affect the PTEN pathway. We found no significant difference in PTEN levels between groups, indicating that radiation did not directly affect PTEN expression. Some radiation alters PTEN signaling pathways. No direct inhibitory effect is shown. PTEN-related mechanisms are possible.

Another parameter examined in our study was TGF-β3 expression, which plays a role in tissue regeneration and fibrosis. DEX is known to prevent fibrotic processes by suppressing TGF-β3 [15]. The Transforming Growth Factor-β (TGF-β) family of cytokines, which includes TGF-Β1, TGF-Β2, and TGF-Β3, plays a role in the pathogenesis of numerous diseases. They are also active in the treatment of cancer and fibrosis [22]. Various treatments can be developed by inhibiting the receptors of these three isoforms. Our study revealed significant staining of TGF-β3 antibodies in the RT group. This suggests that radiation could promote fibrosis in liver tissue. The reduced expression in the treatment groups supports the antifibrotic effects of both amifostine and DEX doses. The observed increase in perisinusoidal fibrosis in the RT-D100 group may reflect a dose-dependent effect of DEX, potentially mediated through upregulation of TGF-β3 signaling. While this change is statistically significant, it should be interpreted cautiously, as further studies would be required to confirm whether it represents a truly adverse or harmful effect. The expression of c-Fos was analyzed to evaluate the impact of radiotherapy on hepatic tissue. The c-Fos protein, an early-response gene implicated in intracellular signaling, is expressed at low levels under standard conditions. It can be swiftly activated following ionizing radiation [23]. In our study, while c-Fos expression increased due to radiotherapy originating from the liver, an interesting increase in c-Fos expression was also noticed in the RT-D200 group. Radiation-generated ROS can activate cellular signaling pathways and stimulate the transcription of c-Fos, an early response gene. This may serve as a marker of oxidative stress in hepatic tissue. The heightened c-Fos immunoactivity detected in the RT group in our investigation may indicate this oxidative signaling. The reduction in c-Fos levels after dexmedetomidine or amifostine therapy illustrates the inhibitory effects of these drugs on oxidative stress [24,25,26]. GAP-43 is actually a marker for muscle regeneration [27]. In our investigation, GAP-43 expression, which is implicated in tissue repair and neural remodeling in non-neuronal tissues, was shown to be diminished in the RT group relative to the control group. A notable reduction in GAP-43 immunoactivity was noted in the DEX-treated groups. GAP-43 expression was found to elevate following amifostine administration [9]. The deterioration of the liver’s intrinsic neural network and the hepatic microenvironment induced by radiation may elucidate the observed decline in GAP-43 levels. The additional lowering of this expression by DEX at elevated doses (200 µg/kg) may be associated with the inhibitory effects of alpha2-adrenergic receptor activation on brain remodeling processes. The elevation of GAP-43 levels by amifostine indicates its beneficial role in tissue regeneration and neurotrophic signaling. These findings indicate that amifostine promotes brain healing, but DEX, despite its antioxidant properties, may inhibit the neural regenerative response. DEX and amifostine treatment did not influence S100β levels, a calcium-binding protein that may be elevated due to cellular stress and oxidative imbalance. The modest rise in the RT group may suggest radiation-induced stress [10]. DEX, which has significant antioxidant and anti-inflammatory properties, can reduce neuroplasticity-related signaling through an α_2_-adrenergic receptor-mediated mechanism [16,28]. Considering that GAP-43 immunoreactivity in the liver represents intrahepatic nerve regeneration, the decreased expression observed in the DEX groups may be associated with suppressed neurotrophic signaling.

The main limitation of our study is that it was conducted only on a male rat model; the effects of gender differences or other species are not yet known. Furthermore, functional results were obtained at the histological, biochemical, and molecular levels and were not extended. Therefore, our study can be considered a preliminary study. The study was conducted only on an animal model (rat); effects on humans cannot be directly inferred. Only certain tissue and molecular parameters were examined, and the study did not include long-term functional effects. The doses and durations of Dexmedetomidine and amifostine administered are limited; different doses or treatment durations may yield different results.

The findings from our study demonstrate the potential of dexmedetomidine and amifostine to reduce radiation- or other stress-induced tissue damage. These results may provide fundamental data for future clinical use of tissue-sparing or neuroprotective treatments in humans. They provide preliminary data suggesting that these agents may have a role in tissue-sparing strategies, particularly in patients undergoing perioperative or radiation therapy.

In summary, immunohistochemical and histopathological methods demonstrate that RT induces specific effects on neuronal remodeling and the cellular stress response in liver tissue. The elevation of GAP-43 levels by amifostine indicates its neuroprotective properties, thereby facilitating tissue regeneration. The antioxidant effects of DEX indicate a complex regulatory role in neural plasticity. This implies that both agents may operate through distinct mechanisms to mitigate radiotherapy-induced liver damage. Future research should explore these pathways at the molecular level.

## 5. Conclusions

In our study, low-dose DEX (100 µg/kg) administration was observed to exhibit more pronounced protective effects on some histological and immunohistochemical parameters (e.g., Ki-67 and c-Fos expression). This may be due to the dose-dependent bidirectional effect of DEX. Low-dose DEX enhances antioxidant defenses through selective activation of α2-adrenergic receptors, while high-dose DEX can suppress cellular metabolism through excessive activation of α2-adrenergic receptors. As a result, oxidative balance may be disrupted and apoptosis may be induced. Although the optimal therapeutic dose for DEX is limited, it is thought to be effective in preserving structural morphology even at low doses. The combined evaluation of histopathological and immunohistochemical results indicated that RT elicited a tissue response marked by notable morphological damage, hepatocyte degeneration, disruption of the sinusoidal architecture, and fibrotic alterations in liver tissue. DEX administration reduced the morphological damage induced by radiation, notably by significantly decreasing the occurrence of pyknotic nuclei, cellular degeneration, and fibrosis scores. Moreover, amifostine facilitated hepatic regeneration by upregulating the expression of GAP-43.

## Figures and Tables

**Table 1:** Histopathological Scoring.

**Periportal Fibrosis** Statistical data regarding periportal fibrosis are shown.					
	**Control**	**RT**	**RT-D100**	**RT-D200**	**RT-A**
Minimum	0.0	0.0	0.0	0.0	1
25% Percentile	0.25	0.0	0.0	0.0	1.25
Median	1	0.0	1	1	2.5
75% Percentile	1	0.75	1.75	1	3
Maximum	1	1	2	2	3
Std. Error of Mean	0.1637	0.1637	0.295	0.25	0.3134
Lower 95% CI	0.3630	−0.1370	0.1773	0.1588	1.509
Upper 95% CI	1.137	0.6370	1.573	1.341	2.991
**Perisinusoidal Fibrosis** Statistical data regarding perisinusoidal fibrosis are shown.					
	**Control**	**RT**	**RT-D100**	**RT-D200**	**RT-A**
Minimum	0.0	0.0	2	0.0	0.0
25% Percentile	0.25	0.0	2	0.0	1
Median	1	1	2.5	1	1.5
75% Percentile	1	1	3	1	2
Maximum	1	2	3	2	2
Mean	0.75	0.75	2.5	0.75	1.375
Std. Deviation	0.4629	0.7071	0.5345	0.7071	0.7440
Std. Error of Mean	0.1637	0.25	0.189	0.25	0.2631
Lower 95% CI	0.363	0.1588	2.053	0.1588	0.753
Upper 95% CI	1.137	1.341	2.947	1.341	1.997
**Vacuolization** Statistical data regarding vacuolization are shown.					
	**Control**	**RT**	**RT-D100**	**RT-D200**	**RT-A**
Minimum	0.0	0.0	2	0.0	0.0
25% Percentile	0.25	0.0	2	0.0	1
Median	1	1	2.5	1	1.5
75% Percentile	1	1	3	1	2
Maximum	1	2	3	2	2
Mean	0.75	0.75	2.5	0.75	1.375
Std. Deviation	0.4629	0.7071	0.5345	0.7071	0.744
Std. Error of Mean	0.1637	0.25	0.189	0.25	0.2631
Lower 95% CI	0.363	0.1588	2.053	0.1588	0.753
Upper 95% CI	1.137	1.341	2.947	1.341	1.997
**Pyknotic nuclei** Statistical data regarding pyknotic nuclei are shown.					
	**Control**	**RT**	**RT-D100**	**RT-D200**	**RT-A**
Minimum	1	2	1	0.0	0.0
25% Percentile	1	2	2	1	0.0
Median	1	3	2	1	0.5
75% Percentile	1.75	3	3	2	1
Maximum	3	3	3	2	1
Mean	1.375	2.625	2.25	1.25	0.5
Std. Deviation	0.744	0.5175	0.7071	0.7071	0.5345
Std. Error of Mean	0.2631	0.183	0.25	0.25	0.189
Lower 95% CI	0.753	2.192	1.659	0.6588	0.05313
Upper 95% CI	1.997	3.058	2.841	1.841	0.9469

## Data Availability

The data presented in this study are available on request from the corresponding author. The data are not publicly available due to ethical restrictions.

## Abbreviations

The following abbreviations are used in this manuscript:

A	Amifostine
AI	Apoptotic index
ALT	Alanine aminotransferase
DEX	Dexmedetomidine
GAP-43	Growth-Associated Protein 43
IR	Ischemia–reperfusion
LDH	Lactate Dehydrogenase
MI	Mitotic index
PTEN	Phosphatase and tensin homolog
RT	Radiotherapy
TT	Total Thiol
TBARS	Thiobarbituric Acid Reactive Substances.
